# Shift Work as a Risk Factor for Future Type 2 Diabetes: Evidence, Mechanisms, Implications, and Future Research Directions

**DOI:** 10.1371/journal.pmed.1001138

**Published:** 2011-12-06

**Authors:** Mika Kivimäki, G. David Batty, Christer Hublin

**Affiliations:** 1 Department of Epidemiology and Public Health, University College London, London, United Kingdom; 2 MRC Centre for Cognitive Ageing and Cognitive Epidemiology, University of Edinburgh, Edinburgh, United Kingdom; 3 Finnish Institute of Occupational Health, Helsinki, Finland

## Abstract

Mika Kivimaki and colleagues discuss new research that shows an association between shift work and the risk of developing type 2 diabetes among nurses.

**Linked Research Article**

This Perspective discusses the following new study published in *PLoS Medicine*:

Pan A, Schernhammer ES, Sun Q, Hu FB (2011) Rotating Night Shift Work and Risk of Type 2 Diabetes: Two Prospective Cohort Studies in Women. PLoS Med 8(12): e1001141. doi:10.1371/journal.pmed.1001141.

An Pan and colleagues examined data from two Nurses Health Studies and found that extended periods of rotating night shift work were associated with a modestly increased risk of type 2 diabetes, partly mediated through body weight.

## 

In Western countries, exposure to traditional occupational hazards—toxic chemicals, temperature extremes, and noise—has been minimized to the extent that they now represent a modest risk to employees' health. The focus has moved to other characteristics of the workplace that may be of importance: work design, organization, management, and their societal contexts. One occupational factor that has potential health implications is shift work. Shift work can be classified in one of two ways: “rotating”, where the employee's hours of work change (e.g., morning, afternoon, and night shift); and “permanent,” where the work pattern may be constant but occupy unusual hours of the day.

That shift work is as common as smoking in Western societies—around one-fifth of workers may be so classified—means that even a modest detrimental impact on health may have important public health implications. Thus, the recent hypothesis that shift work is related to type 2 diabetes (T2D), an increasingly common progressive disorder that can cause a failure of function in vital organs (kidneys, nerves, heart, and blood vessels), merits attention. A new paper in this week's *PLoS Medicine* [1] adds to the evidence base.

There are biologically plausible reasons to anticipate a link between shift work and T2D (Figure 1). Human homeostatic systems have adapted to daily changes in light and dark such that the body anticipates periods of physical exertion and sleep. Circadian clocks, located both in the central nervous system (hypothalamus) and peripheral tissues, regulate this circadian rhythm, thus influencing the function of the endocrine system and gastrointestinal tract [2]. Shift work—which interferes with the normal synchrony between the light-dark cycle, sleeping, and eating—may cause a mismatch of circadian rhythms, triggering a cascade of biological changes that have potential diabetogenic effects. As a result, circadian disruption may accelerate development of T2D in diabetes-prone individuals [3]. Human evidence suggests that insufficient sleep and poor sleep quality—common consequences of shift work—are independent risk factors for the development and exacerbation of insulin resistance [4], and lead to increases in appetite and adiposity [5]. In agreement with this, a recent meta-analysis of eight longitudinal studies showed a strong association between shift work and weight gain, a major risk factor for T2D [6]. Furthermore, behavioral changes potentially associated with shift work, such as reduced physical activity, could independently contribute to biological processes predisposing to T2D.

**Figure 1:**
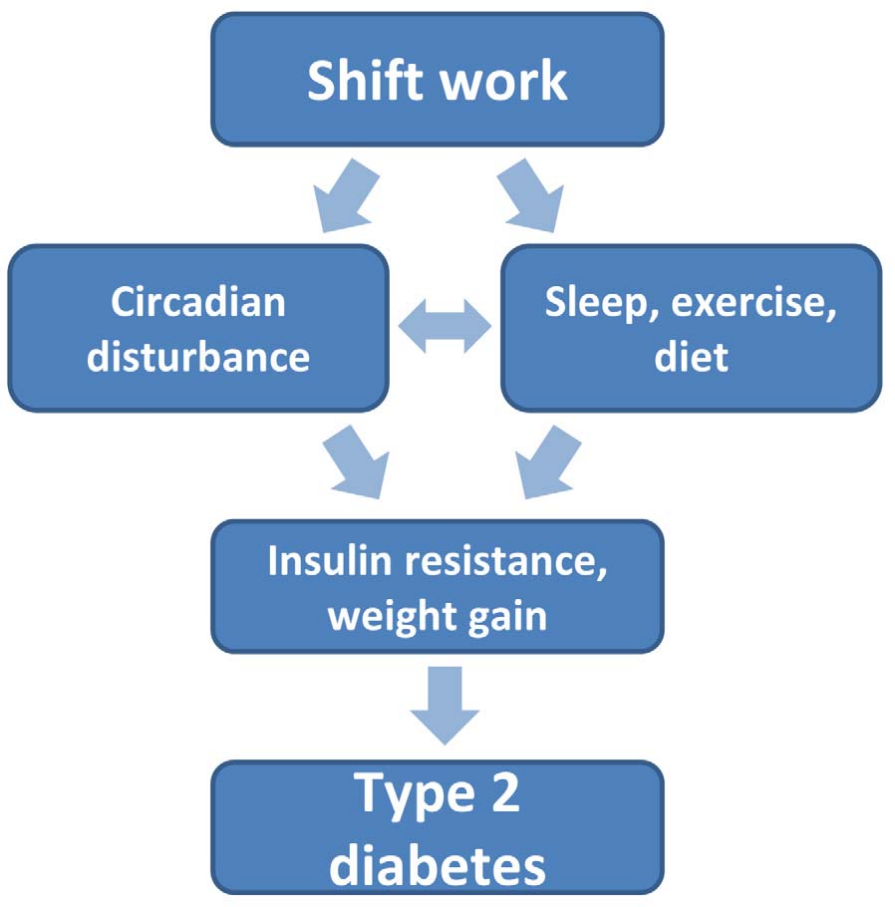
Links between shift work and type 2 diabetes.

## New Findings on Shift Work and Diabetes Risk

In this issue of *PLoS Medicine*, An Pan et al. contribute substantially to this field of research by examining the association between rotating shift work (≥3 nights/month plus days and evenings) and T2D among 177,000 female nurses aged 25–67 at baseline followed up for up to two decades (the Nurses' Health Study) [1]. This large-scale study revealed a graded association between the duration of working life the nurses had been engaged in shift work and risk of developing T2D. Compared with women who reported no shift work, participants with 1–2 years of shift work had a 5% excess risk of T2D, rising to 20% after 3–9 years, 40% after 10–19 years, and almost 60% for ≥20 years.

In observational studies, statistical adjustments are used to assess the role of potential mediating and confounding factors; a marked attenuation in risk estimates relative to the unadjusted estimates suggests that either mediation or confounding is operating. Pan et al. adjusted their analysis for many lifestyle and health-related factors without substantial effect on the shift work–T2D gradient. The only exception was body mass index, where heavy attenuation was seen. This provides support for the hypothesis that the mechanisms underlying the shift work–T2D relation involve weight gain.

These data, which were based on a long follow-up, are in agreement with previous smaller-scale studies on incident T2D [7],[8] and impaired glucose metabolism, a key mechanism underlying the development of T2D [9]. In observational epidemiology, drawing on a single occupational group is often regarded as a major limitation in terms of the generalizability of findings, but this is probably outweighed by the internal validity offered. In prior research of general population samples, which, by definition, capture a range of occupational groups, shift work is more common in people of lower socioeconomic status. It therefore becomes problematic to separate the deleterious impact of shift work (if any) from that of socioeconomic adversity, a strong risk factor for T2D in its own right. This problem was therefore largely avoided in the study of nurses reported by Pan et al.

Another advantage of single occupation studies involves the avoidance of heterogeneity in the explanatory mechanisms between different occupations. The role of physical inactivity as a mediator between shift work and T2D might be more pronounced, for instance, among long-distance truck drivers with limited opportunities to exercise during their journeys. Similarly, in some occupations, shift working is additionally combined with long working hours, a further health risk factor. Thus, focusing on exposed and comparison groups within a single occupation simplifies interpretation.

## Size of the Effect

In this context and in others, the Nurses' Health Study has been highly influential in the field of chronic disease epidemiology; in particular, the use of mailed questionnaires has facilitated the on-going surveillance of a sample of health professionals that is large enough so as to provide highly reliable estimates of risk factor–disease associations. Reliance on self-reports does, however, introduce some uncertainty in the present context. In clinical practice, T2D is ascertained based on elevated glucose levels, and population-based studies using this method suggest that in a substantial proportion of diabetic people the condition is hidden; that is, they are unaware of its existence. By using self-reports, Pan et al. measured only diagnosed T2D. This does not introduce bias if the proportion of undiagnosed T2D is non-differential with respect to the exposure—shift and non-shift workers—but this is not necessarily the case, and therefore confirmation from future studies with blood-based definition of T2D are needed.

Furthermore, selection processes could have artificially *inflated* the association between shift work and T2D because unmeasured individual differences, such as personality traits, could affect both the extent to which a nurse manages to find a work schedule more convenient than shift work and is able to maintain a healthy lifestyle that protects against T2D.

These limitations notwithstanding, the study by Pan et al. probably represents the most accurate estimate of the shift work–T2D association available to date, suggesting this effect is comparable in size to that of work stress on coronary heart disease and larger than the effect of work stress on T2D [10].

## Clinical Implications

We are increasingly residing in a “24/7” society; thus, the option to eradicate shift working is not realistic. If the observed association between rotating shift work and T2D is causal, as it may be, additional efforts to prevent T2D among shift workers through promotion of healthy lifestyles, weight control, and early identification and treatment of prediabetic and diabetic employees are needed. Some modifications to shift work itself might also be feasible. Rotating shift work comprises a range of alternative schedule patterns, such as backward- and forward-rotating shift systems, and the proportion of night and early morning shifts varies. Future studies should address these variations and identify patterns that minimize T2D risk, ideally through large-scale randomized trials that would provide insights into causality.

## Abbreviations

T2D: type 2 diabetes
